# The safety and efficacy of applying a high-current temporal interference electrical stimulation in humans

**DOI:** 10.3389/fnhum.2024.1484593

**Published:** 2024-11-29

**Authors:** Yan Wang, Ginger Qinghong Zeng, Mengmeng Wang, Mingsong Zhang, Chuangchuang Chang, Qiongwei Liu, Keqing Wang, Ru Ma, Ying Wang, Xiaochu Zhang

**Affiliations:** ^1^School of Mental Health, Bengbu Medical University, Bengbu, China; ^2^Division of Life Science and Medicine, University of Science and Technology of China, Hefei, China; ^3^Application Technology Center of Physical Therapy to Brain Disorders, Institute of Advanced Technology, University of Science and Technology of China, Hefei, China; ^4^Anhui Provincial Stereotactic Neurosurgical Institute, Anhui Province Key Laboratory of Brain Function and Brain Disease Hefei, Hefei, China; ^5^Department of Psychology, School of Humanities and Social Science, University of Science and Technology of China, Hefei, China; ^6^Biomedical Sciences and Health Laboratory of Anhui Province, University of Science and Technology of China, Hefei, China

**Keywords:** high-current TI electrical stimulation, safety, efficacy, noninvasive brain stimulation, transcranial electrical stimulation, motor function

## Abstract

**Background:**

Temporal interference electrical stimulation (TI) is promise in targeting deep brain regions focally. However, limited electric field intensity challenges its efficacy.

**Objective:**

This study aimed to introduce a high-current TI electrical stimulation protocol to enhance its intensity and evaluate its safety and efficacy when applied to the primary motor cortex (M1) in the human brain.

**Methods:**

Safety assessments included a battery of biochemical and neuropsychological tests (NSE, MoCA, PPT, VAMS-R, and SAS measurements), 5-min resting-state electroencephalography (EEG) recordings before and after 30-min high-current TI electrical stimulation sessions (20 Hz, 70 Hz, sham). Adverse reactions were also documented post-stimulation. Efficacy evaluations involved two motor tasks, the simple reaction time (SRT) task and the one-increment task, to investigate the distinct contributions of beta (20 Hz) and gamma (70 Hz) oscillations to motor functions.

**Results:**

Biochemical and neuropsychological tests revealed no significant differences between the groups. Additionally, no epileptic activities were detected in the EEG recordings. In the one-increment task, 20 Hz stimulation delayed participants’ reaction time compared to the 70 Hz and sham groups. Conversely, in the SRT task, 70 Hz stimulation exhibited a tendency to enhance participants’ performance relative to the sham group.

**Conclusion:**

The proposed high-current TI electrical stimulation is both safe and effective for stimulating the human brain. Moreover, the distinct effects observed in motor tasks underscore the dissociative roles of beta and gamma oscillations in motor functions, offering valuable insights into the potential applications of high-current TI electrical stimulation in brain stimulation research.

## 1 Introduction

Temporal interference (TI) electrical stimulation, a novel transcranial electrical stimulation (tES) method, utilizes two pairs of scalp electrodes to deliver high-frequency electric fields with slight frequency differences, generating a low-frequency envelope that modulates brain activity (Grossman et al., 2017). Compared with conventional tES, TI electrical stimulation provides greater precision in targeting capabilities for deep brain structures while reducing its impact on superficial and peripheral regions (Liu et al., 2022; Guo et al., 2023; Zhu and Yin, 2023). While Grossman proposed the low-pass filtering characteristics of neural membranes as the reason for neurons reacting to TI electrical stimulation (Palmer et al., 1999; Hutcheon and Yarom, 2000), other studies have suggested that TI electrical stimulation involves a signal rectification process mediated by ion channels (Mirzakhalili et al., 2020) and subthreshold modulation also plays a significant role (Howell and McIntyre, 2021). Further research is needed to fully understand the mechanisms of TI electrical stimulation.

TI electrical stimulation has proven successful in activating the primary motor cortex (M1) in rats, eliciting hippocampal and superior colliculus responses in mice, and inducing movements corresponding to envelope frequencies (Grossman et al., 2017; Song et al., 2021; Zhang Z. et al., 2022). Computational modeling studies utilizing human head model demonstrated reduced activation of superficial areas compared to transcranial alternating current stimulation (tACS) when targeting deep brain regions with TI simulation (Huang and Parra, 2019; Rampersad et al., 2019). Human research has further validated the efficacy of TI electrical stimulation, showing its positive impact on motor function (Ma et al., 2022; Zhu et al., 2022), enhancement of hippocampal function (Violante et al., 2023), and improvements in behavioral performance and striatal activities, particularly among healthy elderly individuals (Wessel et al., 2023). Notably, in another study, the researchers found that TI simulation did not modulate alpha-band brain oscillations compared to the sham group. This may be due to the low current used in this study (von Conta et al., 2022). Details of TI electrical stimulation studies in human are presented in the Supplementary Table 2. The efficacy of TI electrical stimulation in diverse applications shows promise for non-invasive deep brain stimulation in treating neurological disorders.

The safety and efficacy of employing TI electrical stimulation (zero-to-peak 1 mA in a single channel) in human studies has been evaluated. Ma et al. applied TI electrical stimulation (1 mA, 20/70 Hz, 30 min) with 2,000-Hz carrier frequency to the left primary motor cortex in healthy adults for the first time and demonstrated that 20 Hz TI electrical stimulation enhanced the reaction time performance of the serial reaction time task (SRTT), while 70 Hz TI electrical stimulation had a promoting effect on the performance of the random reaction time task (RRTT) and excitability of the primary motor cortex (Ma et al., 2022). In 2022, researchers applied TI electrical stimulation to the primary motor cortex of healthy subjects, and conducted a series of physiological and neuropsychological tests on subjects before and after stimulation. Results showed that the approach used in Ma et al. did not cause physiological or neuropsychological changes in subjects compared with sham group, firstly supported that TI electrical stimulation is safe and tolerable for humans (Piao et al., 2022).

However, the current application of tES generally is yielding relatively weak effect, with one significant reason being the low stimulation intensity. Research indicates a close relationship between the stimulation intensity and its effectiveness (Moliadze et al., 2012; Antonenko et al., 2019; Kasten et al., 2019; Steinmann et al., 2022; Shan et al., 2023). To adhere to safety and comfort standards set for conventional tES (Bikson et al., 2016; Antal et al., 2017), previous TI electrical stimulation studies also used a stimulation intensity range of 0–4 mA (Ma et al., 2022; Piao et al., 2022; Zhang Y. et al., 2022; Zhu et al., 2022; Violante et al., 2023; Wessel et al., 2023). The stimulation intensity of conventional tES is constrained by the participants’ discomfort caused by skin sensation, though higher intensities are considered safe (Fertonani et al., 2015; Bikson et al., 2016; Antal et al., 2017). On the contrast, it is feasible to use high current intensity in TI as it causes weaker adverse reactions and skin sensation than tACS when applying the same current intensity (Turi et al., 2013), which benefits from the high carrier frequency of TI.

The perception of skin sensation induced by tES is closely associated with the frequency of stimulation, with higher frequencies exhibiting lower sensation sensitivity (Turi et al., 2013; Zeng et al., 2019; Hsu et al., 2021). Previous tACS studies have demonstrated that it was safe to apply a current of 10 mA using 5 kHz transcranial alternating currents (tACS) on M1 of humans (Kunz et al., 2017). Another study applied 15 mA (zero-to-peak) tACS on patients with depression (Wang et al., 2022), which yielded stronger therapeutic effects compared with the low-current tACS while being proven safe, well-tolerated without causing impairment or cognitive defects. Similar situation was founded when treating chronic insomnia by the 15 mA tACS (Zhu et al., 2024). The absence of significant adverse reactions to such high current is attributed to the utilization of a high frequency. Therefore, TI electrical stimulation, which utilizes high-frequency current signals, has the potential to enhance the current intensity to obtain a more potent brain conditioning effect without causing excessive skin discomfort.

In line with the tACS studies delineated above (Wang et al., 2022; Zhu et al., 2024), we proposed a high-current TI electrical stimulation approach in which each pair of electrodes delivers a stimulation intensity of up to 15 mA (zero-to-peak) in a single channel. However, it is important to note that a higher TI carrier frequency does not necessarily yield better results (Vieira et al., 2024). One drawback is that a high frequency of TI electrical stimulation results in a weaker electric field within the brain, as the conductivity of biological tissue exhibits frequency dependence (Hasgall et al., 2015). Additionally, the high frequency of TI electrical stimulation causes more current to be diverted through the skin, muscle, and bone (Vöröslakos et al., 2018). Furthermore, increasing the TI electrical stimulation frequency also raises the activation threshold of neurons (Gomez-Tames et al., 2021). In order to adhere to the high current proposed, we conducted a skin sensation test to meet comfort standards and at the same time made a balance between the frequency and intensity by selecting a smaller frequency (i.e., 20 kHz) as to maximize the potential effect.

The current study would assess the safety and efficacy of the high-current TI electrical stimulation on M1 in healthy individuals. The safety assessments encompass a serials of biochemical and neuropsychological tests, with comprehensive evaluation of potential neuronal damage, cognitive function, emotional state, adverse reactions, and seizure risks. Envelope frequencies of 20 and 70 Hz are employed to investigate their distinct roles in motor tasks, with prior research indicating gamma oscillations (70 Hz) influence movement speed and initiation (Cheyne et al., 2008; Joundi et al., 2012; Moisa et al., 2016), while beta oscillations (20 Hz) are linked to motor learning (Krause et al., 2016; Espenhahn et al., 2019, 2020). Through motor task assessments pre- and post-stimulation, we hypothesized a differential effects of 70 and 20 Hz high-current TI electrical stimulation on motor performance.

## 2 Materials and methods

### 2.1 Participants

Ninety healthy adult participants were recruited with informed consent and randomly assigned to three groups: two active groups (20 Hz/70 Hz) and one sham group. After excluding two participants due to instrument malfunction and procedural errors, the final analysis included 88 participants (20 Hz group: *N* = 29, 14 females, age range: 18–27 years, mean age ± SD: 22.24 ± 2.25 years, mean education level ± SD: 16.41 ± 1.55 years; 70 Hz group: *N* = 30, 15 females, age range: 19–30 years, mean age ± SD: 22.97 ± 2.04 years, mean education level ± SD: 16.90 ± 1.37 years; sham group: *N* = 29, 13 females, age range: 18–28 years, mean age ± SD: 22.59 ± 3.05 years, mean education level ± SD: 16.14 ± 2.10 years). No significant differences in ages and education levels were observed among the groups [Fs(2,85) = 0.079 and 0.631, ps = 0.535 and 0.924]. All participants were right-handed as assessed with the Edinburgh Handedness Inventory (Oldfield, 1971) and had normal or corrected-to-normal vision. No participants reported a history of craniotomy or injury to the head, personal or family history of neurological or psychiatric disease, metal implants or implanted electronic devices, skin sensitivity or use of medicine during the experiment. For safety reasons, any participant who was pregnant or could be pregnant was rejected. This study was approved by the Human Ethics Committee of the University of Science and Technology of China (IRB Number: 2022KY275).

### 2.2 Experimental procedure

**Figure 1:**
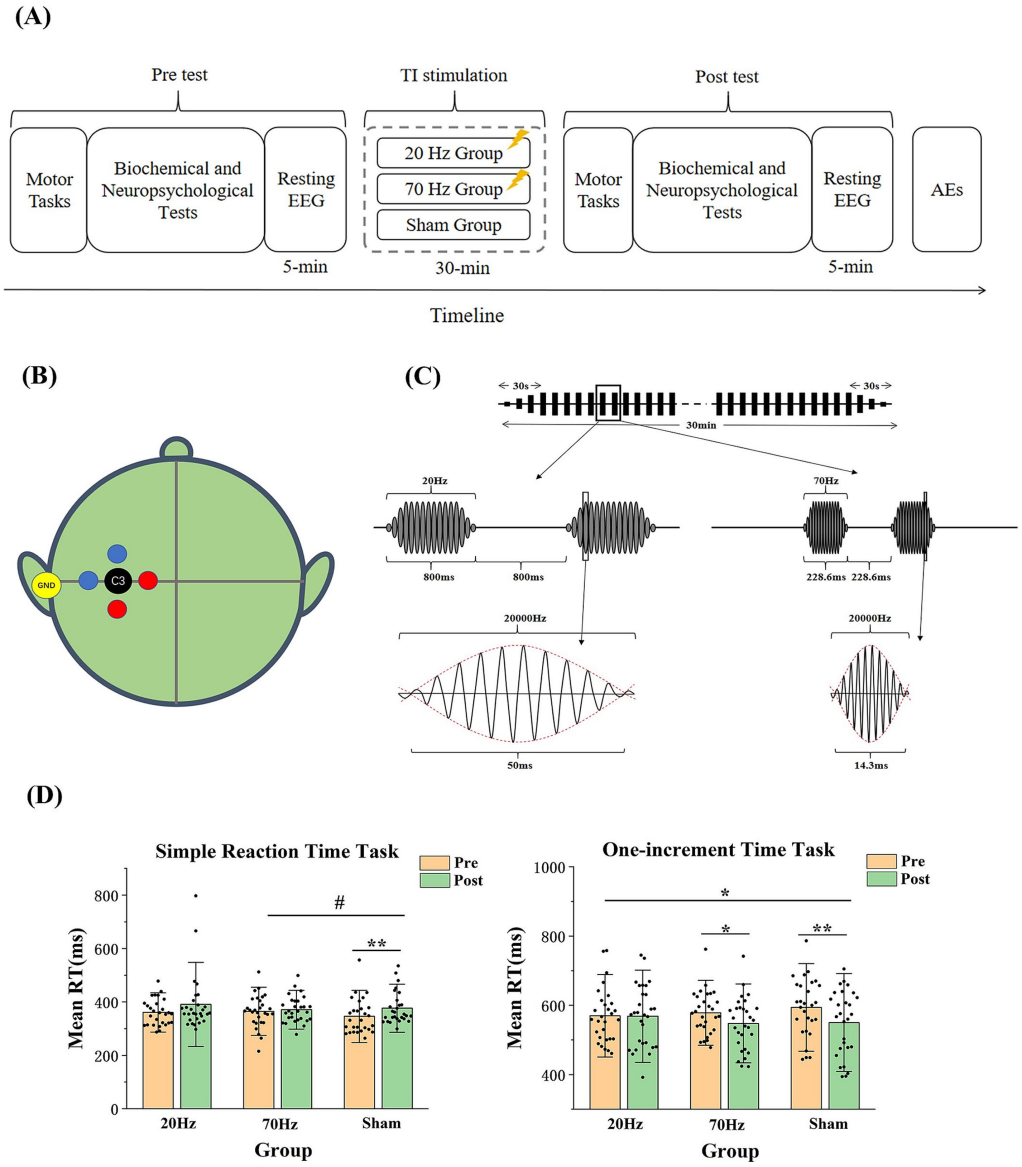
(A) Experimental Procedure. (B) Locations of the stimulation electrodes and the target. Two pairs of electrodes are placed 3 cm away from C3 (one pair in red and the other pair in blue), with the yellow ground electrode placed at the left mastoid behind the ear. The position of the C3 electrode is located at 30% of the distance along the line from the left to the right preauricular points. (C) Schematic diagram of the high-current TI electrical stimulation. More specifically, each stimulation is composed of 16 envelopes with 3 ones rising at the beginning and falling at the end. As one envelope takes 50 ms in the 20 Hz session and 14.3 ms in the 70 Hz session, the stimulation duration is 800 ms in the 20 Hz session and 228.6 ms in the 70 Hz session. (D) Results of the mean reaction time in the simple reaction time task and the one-increment task. RT is reaction time. Error bars represent SEM. * indicates *p* < 0.05; ** indicates *p* < 0.01; # indicates *p* = 0.059.

Participants underwent a 30-min session of active (20 or 70 Hz) or sham TI electrical stimulation in a single-blind parallel design. Motor tasks, biochemical and neuropsychological tests, and a 5-min resting-state EEG recording (eye-closed) were conducted before and after stimulation. A subjective questionnaire on adverse reactions (AEs) was administered post-stimulation (see Figure 1A).

### 2.3 The motor tasks

Two motor tasks (Miller et al., 1991) were performed: the simple reaction time task (SRT) and the one-increment task, assessing basal reaction time and response to sequential number stimuli, respectively.

In the simple reaction time task (SRT; Miller et al., 1991), the participants were asked to press the space bar as soon as they saw a number from 0 to 9 appearing in the middle of the screen to measure the basal reaction time. Random inter-stimulus-interval (ISI) ranges from 1,000 to 5,000 ms. The task consists of 4 practice trials and15 test trials.

In the one-increment task (Miller et al., 1991), the participants were asked to press the space bar only when they see two numbers in sequence with one increment. For instance, if they see the number of 3 followed by the number of 4 or the number of 6 followed by the number of 7, the space bar was required to be pressed. The number was presented for 100 ms. The ISI was set as 800 ms. It consisted of 100 trials with 20 target stimuli. Before the formal task, 19 practice trials with 4 target stimuli were presented with stimulus duration of 400 ms and ISI of 1,000 ms.

### 2.4 Biochemical and neuropsychological tests

Based on the previous safety evaluation conducted by Piao et al. (2022) on low-current TI electrical stimulation, the subsequent biochemical and neuropsychological tests were carried out. The Purdue Pegboard Test (PPT; Tiffin and Asher, 1948) evaluates finger and hand dexterity using a board with holes for pin placement. The test board consists of two parallel rows of 25 holes each, with pins (pegs) positioned at the top corners on the right and left sides. The middle cups hold collars and washers. During the initial three subtests, participants aim to place the maximum number of pins into the holes within a 30-s interval, starting with their dominant hand, followed by their nondominant hand, and finally using both hands simultaneously. In the fourth subtest, individuals are required to use both hands alternately to create ‘assemblies’ within a 1 min. The assembly sequence involves placing a pin, followed by a washer, a collar, and another washer. Each subtest is conducted three times to ensure the reliability of the results.

The Montreal Cognitive Assessment (MoCA; Nasreddine et al., 2005) screens for cognitive impairment across various domains, including attention and concentration, executive function, memory, language, visual-structural skills, conceptual thinking, computation, and orientation. The maximum score on the test is 30. Two versions of the MoCA were alternated between pre- and post-stimulation across participants.

Serum neuron-specific enolase (NSE; Steinhoff et al., 1999) measures neuronal damage, with blood samples analyzed for NSE levels. The blood of the participants was drawn by nurses in the Anhui Provincial Hospital. Blood samples in the post-test were taken half an hour after the electrical stimulation. The blood samples were centrifuged after it has been drawn and the test of the blood samples was carried out in ADICON Medical Laboratory in Hefei by chemiluminescence method. In the test report, the NSE value and a reference value of the normal range (≦16.5 μg/L) are listed.

The Visual Analog Mood Scale (VAMS-R; Kontou et al., 2012) and a self-assessment scale (SAS; Folsten and Luria, 1973) gauge mood and cognitive states. AEs (Brunoni et al., 2011) post-stimulation are assessed using a questionnaire of eight items including itching, headache, burning sensation, warmth, stinging, metallic taste, fatigue, dizziness, nausea, and sensitivity to light, with intensity ratings from 0 to 4 indicating none, mild, moderate, considerable, and intense, respectively.

### 2.5 EEG recording

Resting-state EEG was recorded with OpenBCI, which is an open source EEG acquisition device (Cardona-Álvarez et al., 2023). OpenBCI consists of an 8-channel amplifier (3IT_EEG OBCI Kits), a 3D-printed electrode cap, and dry EEG comb electrodes. An USB dongle enables communication between the amplifier and the computer, and the software OpenBCI_GUI is used to present and record EEG signals in real time. We recorded EEG signals from eight channels (Fp1, Fp2, T3, T4, T5, T6, O1, and O2), with reference electrodes on the earlobes. Electrodes were placed according to the EEG international 10–20 system. The sampling rate is 500 Hz. Participants were asked to keep their eyes closed and head still during the 5-min EEG recording. EEG signals were monitored online by a clinical doctor to check any epileptic seizure. Epileptic activities were also checked offline by an automated software (Encevis, AIT Austrian Institute of Technology GmbH, https://www.encevis.com/), which has been validated in multiple clinical datasets (Fürbass et al., 2017; Rommens et al., 2018).

### 2.6 The skin sensation test

**Figure 2:**
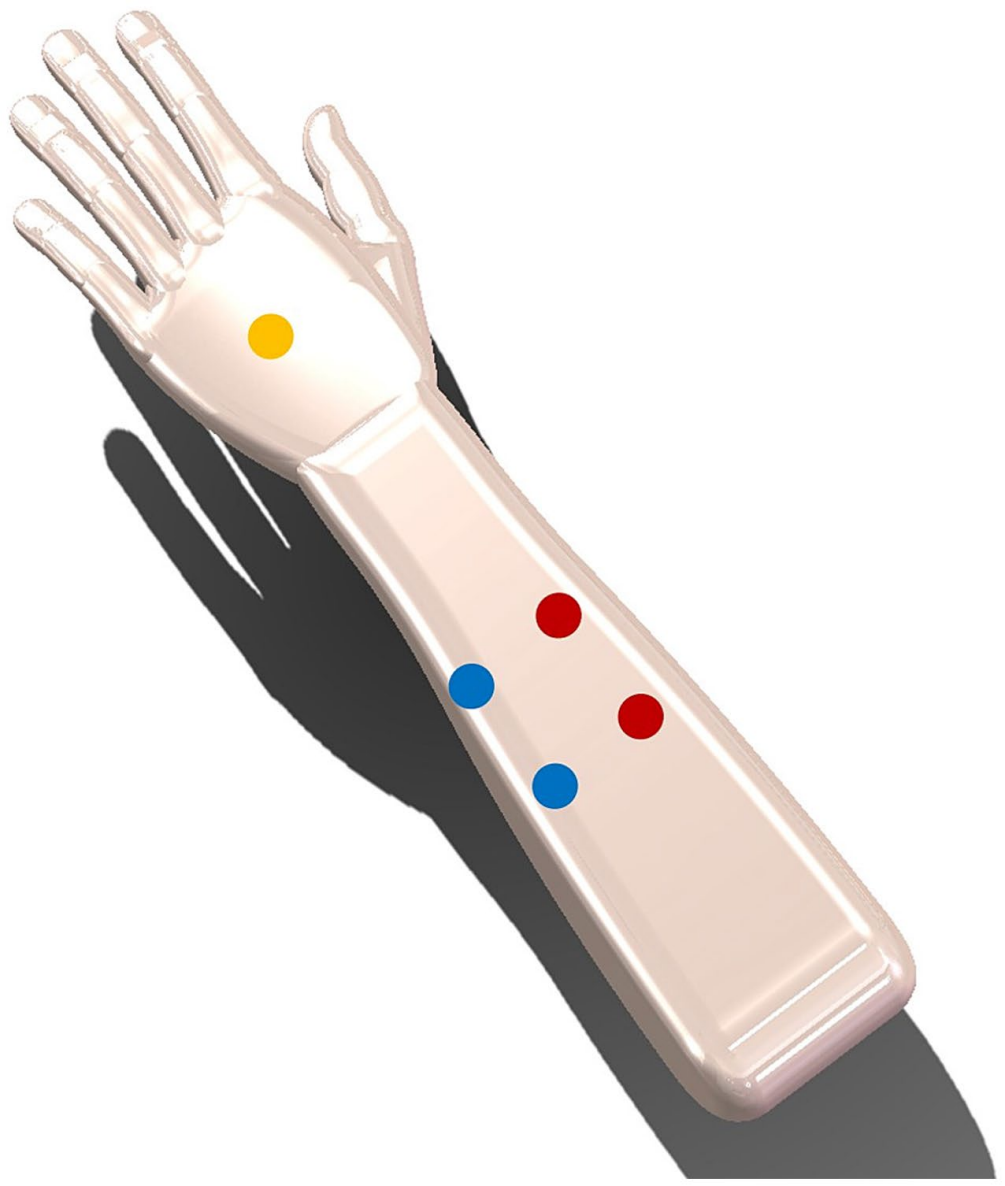
Two pairs of electrodes are placed 3 cm away from the centre (one pair in red and the other pair in blue), with the yellow ground electrode placed at the back of the hand.

The skin sensation test was used to select a proper carrier frequency and current intensity, which could balance both the skin sensation and stimulation effectiveness. The skin sensation test in this study was based on a previous study by Hsu et al. (2021). In the skin sensation test, the carrier frequencies of 5, 10, 20, and 50 kHz and the current intensities of 5, 7.5, 15, 12.5, and 15 mA (zero-to-peak) were used. Ten participants (4 females, mean age ± SD: 25.6 ± 3.69) were randomly exposed to the above 20 (4 carrier frequencies × 5 current intensities) kinds of TI electrical stimulation on the left forearm (see Figure 2). Each stimulation lasted for 1 min with 30 s-rise at the beginning and 30 s-fall at the end of the stimulation. Following each stimulation, participants were instructed to complete skin sensation assessments related to intensity, actinesthesia, pressure, tingling, vibration, muscle contraction, and pain. The intensity perception was evaluated on a 5-likert scale, “No Sensation,” “Mild,” “Moderate,” “Severe,” and “Extreme,” recorded as discrete values of 1, 2, 3, 4, and 5, respectively. For items of actinesthesia, pressure, tingling, vibration, and contraction, they were evaluated on a visual analog scale with 0% indicating no sensation and 100% indicating the strongest sensation. The pain level was rated from 1 to 10, with 1 indicating no pain, 5 indicating moderate pain and 10 indicating worst pain.

The findings indicated that the skin sensation was mild during the high-current TI electrical stimulation with a carrier frequency of 20 kHz and a current intensity of 15 mA (zero-to-peak), meeting the comfort criteria (intensity perception less than level 3, discomfort less than 50%, pain less than level 5; see Table 1). The skin sensation results of 10 and 5 kHz is too strong to use as the carrier frequency of high-current TI electrical stimulation. Finally, although the subjects’ skin felt weaker at the 50 kHz carrier frequency, consider that a drawback is that the high frequency of TI electrical stimulation leads to weaker electric field in the brain (Vieira et al., 2024). The 50 kHz carrier frequency may deserve weaker effect on brain activity than the 20 kHz carrier frequency. For overall consideration, 20 kHz is the most appropriate.

**Table 1:** The results of the skin sensation test.

Frequency (kHz)	Intensity (mA)	Intensity perception	Actinesthesia	Pressure	Vibration	Tingling	Contraction	Pain
5	5	3.10 ± 0.88	8.0	33.6	30.0	19.0	38.5	2.50 ± 1.43
5	7.5	3.80 ± 0.92	21.5	53.6	36.0	28.5	44.6	3.60 ± 2.27
5	10	3.78 ± 0.67^*^	29.0	45.4	42.9	27.2	55.6	4.11 ± 2.89^*^
5	12.5	4.33 ± 1.00^*^	37.0	52.6	44.1	27.9	58.2	4.33 ± 3.16^*^
5	15	4.78 ± 0.44^*^	31.0	62.4	48.0	28.0	69.8	5.33 ± 3.12^*^
10	5	1.70 ± 0.82	2.0	8.6	7.5	14.0	8.1	1.10 ± 0.32
10	7.5	2.50 ± 0.97	12.0	19.2	12.5	19.6	23.4	1.90 ± 1.29
10	10	3.00 ± 0.94	9.0	31.5	34.0	23.6	37.7	2.80 ± 1.81
10	12.5	3.11 ± 0.60^*^	12.0	34.3	31.6	19.0	33.1	3.33 ± 2.00^*^
10	15	3.90 ± 0.74	29.4	39.4	39.3	28.9	45.8	4.20 ± 2.30
20	5	1.10 ± 0.32	0.2	0.0	0.2	5.8	0.0	1.00 ± 0.00
20	7.5	1.60 ± 0.70	7.5	11.9	3.2	11.9	2.0	1.30 ± 0.48
20	10	2.00 ± 0.94	4.0	18.4	8.5	14.4	9.0	1.40 ± 0.97
20	12.5	2.50 ± 0.71	4.0	25.9	17.0	13.4	23.2	1.70 ± 0.95
**20**	**15**	**2.70 ± 0.82**	**6.0**	**36.1**	**27.0**	**15.0**	**32.1**	**1.90 ± 1.00**
50	5	1.00 ± 0.00	0.0	0.0	0.0	0.5	0.0	1.00 ± 0.00
50	7.5	1.10 ± 0.32	3.0	3.0	0.0	0.0	0.0	1.10 ± 0.32
50	10	1.00 ± 0.00	0.0	0.0	0.0	0.0	0.0	1.00 ± 0.00
50	12.5	1.10 ± 0.32	0.0	3.6	3.0	0.0	1.0	1.00 ± 0.00
50	15	1.20 ± 0.42	2.0	4.6	0.0	0.0	1.0	1.10 ± 0.32

The intensity perception was evaluated on a 5-likert scale with 1 indicating no sensation and 5 indicating extreme. For items of actinesthesia, pressure, tingling, vibration, and contraction, they were evaluated on a visual analog scale with 0% indicating no sensation and 100% indicating the strongest sensation. The pain was evaluated on a 10-likert scale with 1 indicating no sensation and 10 indicating extreme. ^*^: One subject discontinued due to inability to withstand the electrical stimulation. The bold values are the skin sensation brought by TIS with a carrier frequency of 20000 Hz and a current intensity of 15 mA (zero-to-peak).

### 2.7 High-current temporal interference stimulation

Similar to our previous study (Ma et al., 2022; Piao et al., 2022), the high-current TI electrical stimulation was applied using customized stimulators and five circular Ag-AgCl electrodes with a radius of 1.2 cm. The electrode placement was shown at Figure 1B. Stimulation intensity (zero-to-peak 15 mA in a single channel) and carrier frequency (20 kHz) were standardized based on the skin sensation test. Monitoring included a mobile app for real-time current and voltage value and thermocouples to track skin-electrode interface temperatures.

The active stimulation, lasting 30 min at 20 Hz (20,000 and 20,020 Hz) or 70 Hz (20,000 and 20,070 Hz), includes a 30-s rise and fall at the beginning and end. A 50% duty cycle was employed to reduce cumulative stimulation time, alternating between on-and-off states (Figure 1C). Computational modeling predicted skin-to-brain current density ratios ranging from 10:1 to 400:1 (Bikson et al., 2016). Brain current density from the high-current TI electrical stimulation was calculated at 1.4 A/m^2^, significantly lower than the injury threshold of 6.3 A/m^2^ (Bikson et al., 2016; Antal et al., 2017; detailed calculation formulas provided in the Supplementary material). In contrast, sham stimulation involves a 60-s rise and fall at the start. Participants were instructed to relax with eyes open during TI electrical stimulation.

### 2.8 Validation of safety and efficacy using agar tissue phantom

**Figure 3:**
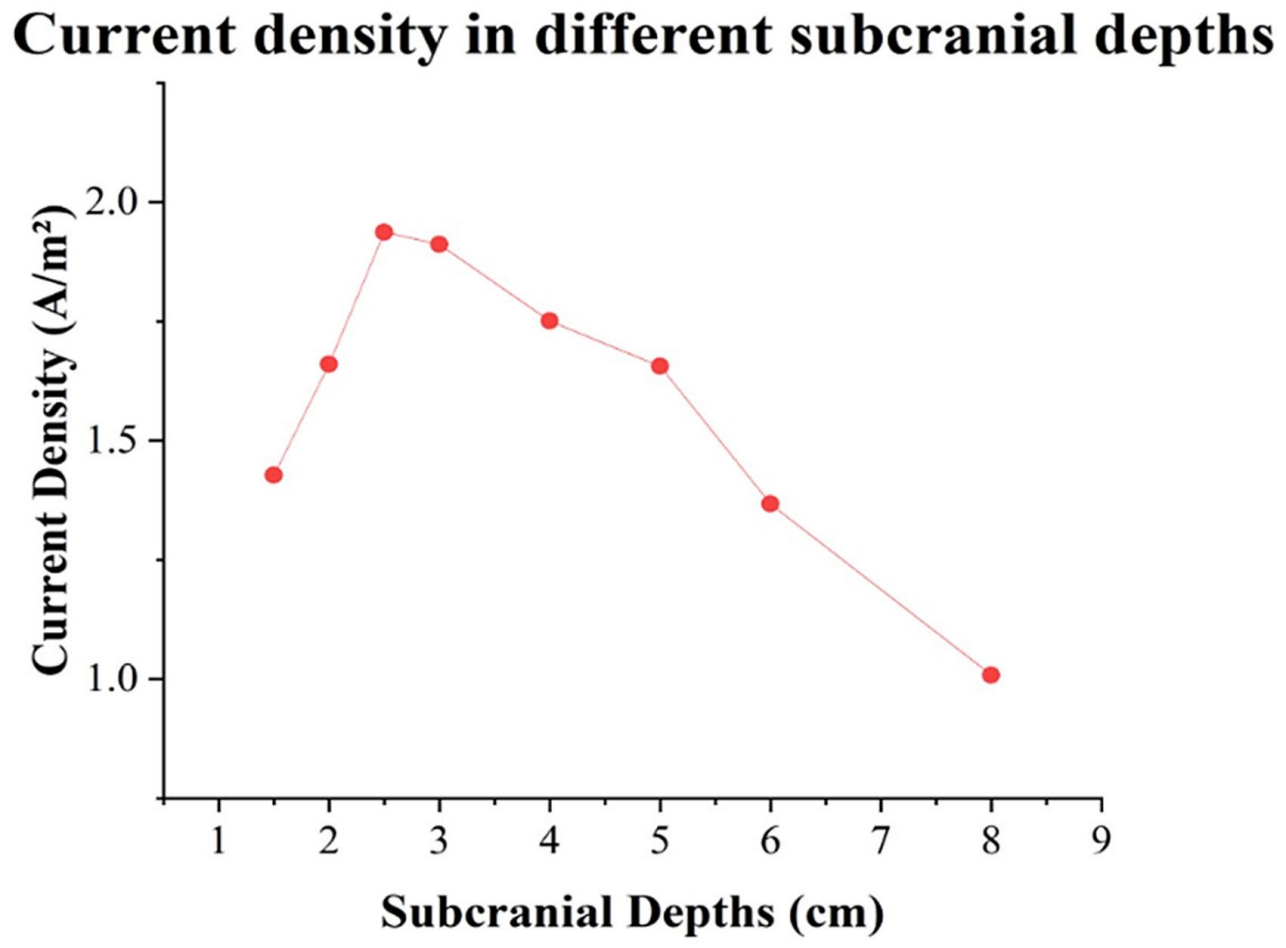
Results of the current densities in different subcranial depths in the midline of brain parenchyma.

To validate the safety and efficacy of the high-current TI electrical stimulation at a physics level, Agar phantoms mimicking diverse brain tissues including scalp, skull, cerebrospinal fluid, and brain parenchyma via injection modeling (Bennett, 2011; Kandadai et al., 2012) were created using different NaCl concentrations and Agar doping ratios (see Table 2). The high-current TI electrical stimulation (intensity: 15 mA (zero-to-peak) in a single channel, carrier frequency: 20 kHz, envelope frequency: 20 Hz) was then applied to the phantom to assess the current density under the four electrodes and within the brain parenchyma at various depths (1.5, 2, 2.5, 3, 4, 5, 6, and 8 cm) were measured by an oscilloscope (RIGOL, DHO4404, China; see Supplementary Figure 2A). Results showed that the current density induced by the high-current TI electrical stimulation under the stimulation electrodes ranged from 1.64 to 1.85 A/m^2^, below the injury threshold of 6.3 A/m^2^. The peak of the interferential electric field envelope magnitude was deeper within the brain parenchyma, resembling an inverted U shape (see Figure 3). Additional details on the methodology and outcomes can be found in the Supplementary material.

**Table 2:** Configuration table of Agar phantom.

	Agar concentration (g/L)	NaCl concentration (g/L)	Conductivity (S/m)
Scalp	30.00	2.28	0.475
Skull	35.00	0.00	0.041
Cerebrospinal fluid	30.00	9.75	1.619
Brain parenchyma	30.00	0.61	0.209

### 2.9 Statistical analysis

Statistical analyses were conducted using IBM SPSS Statistics 26.0 (IBM Corp, Armonk, NY, USA) and MATLAB 2013a (MathWorks, Natick, MA, USA). Participants with reaction times deviating more than 3 standard deviations from the mean in the simple reaction time task were excluded, resulting in 86 participants (20 Hz group: *n* = 29; 70 Hz group: *n* = 29; sham group: n = 28) for final analysis in motor tasks. Differences were evaluated using a 2×3 mixed ANOVA with the time (pre vs. post) as the within-subject factor and the group (20 Hz vs. 70 Hz vs. sham) as the between-subject factor. Follow-up t-tests were conducted if necessary. Group differences in AEs were examined using the chi-square test.

## 3 Results

### 3.1 Safety evaluation

For the NSE, MoCA, PPT, VAMS-R, and SAS measurements, no significant interaction effects of the group and the time were found except for the calmness in the SAS. More details are given in Table 3. The follow-up t tests of the calmness showed a significant decrease in the 20 Hz group [*t*(28) = 2.117, *p* = 0.043, Cohen’s d (*d*) = 0.074], but not in the 70 Hz and sham groups [70 Hz: *t*(29) = 1.649, *p* = 0.110, *d* = 0.301; sham: *t*(28) = −1.440, *p* = 0.161, *d* = 0.267]. For the main effects of the group, there were no significant effects in all measurements except for the NSE and MoCA. For the main effects of the time, most of the measurements were significant except for the MoCA, subitems of sad, happy, and angry in the VAMS-R, subitems of calmness and fatigue in the SAS. After the stimulation, the NSE value was decreased; the performances in PPT were improved; participants felt less confused, afraid, tense, concentrated, energetic, decreased visual perception, and more tired. More details are shown in Supplementary Table 3.

**Table 3:** The statistical results of biochemical and neuropsychological measurements.

Measurements (Range or Unit)	20 Hz group (mean ± SD)		70 Hz group (mean ± SD)		Sham group (mean ± SD)		Statistical results	
	Pre	Post	Pre	Post	Pre	Post	*F*	*p*
MoCA (0–30)	27.48 ± 2.18	27.76 ± 2.03	27.79 ± 1.95	27.97 ± 1.90	26.48 ± 2.60	26.79 ± 1.72	0.03	0.97
NSE (μg/L)	12.68 ± 5.06	11.32 ± 2.98	12.82 ± 2.61	11.52 ± 2.36	15.76 ± 3.85	14.07 ± 3.60	0.30	0.74
PPT (times)								
Right hand	16.18 ± 1.61	16.69 ± 1.67	16.15 ± 1.31	17.14 ± 2.12	16.08 ± 1.26	16.68 ± 1.19	1.07	0.35
Left hand	14.32 ± 1.62	15.22 ± 1.90	14.70 ± 1.67	15.39 ± 1.65	14.59 ± 1.52	14.98 ± 1.42	1.77	0.18
Both hands	25.15 ± 3.22	26.38 ± 2.94	25.17 ± 3.13	26.28 ± 2.85	25.28 ± 2.62	26.05 ± 2.90	0.47	0.63
Assembly	40.84 ± 6.71	44.28 ± 6.52	39.03 ± 6.67	43.83 ± 6.88	37.95 ± 6.48	40.98 ± 7.45	1.87	0.16
VAMS-R (0–100)								
Sad	5.52 ± 10.77	3.64 ± 9.06	5.07 ± 14.93	3.04 ± 8.55	4.86 ± 14.93	4.59 ± 12.03	0.57	0.57
Confused	13.69 ± 23.03	8.29 ± 16.96	12.10 ± 14.02	3.48 ± 8.44	12.57 ± 15.58	6.69 ± 11.76	0.19	0.83
Afraid	7.03 ± 12.58	1.89 ± 4.67	7.93 ± 10.89	2.89 ± 7.17	6.00 ± 8.48	3.14 ± 8.05	0.84	0.44
Happy	34.90 ± 29.12	32.96 ± 30.47	29.33 ± 27.71	29.78 ± 28.87	29.69 ± 32.74	27.86 ± 33.30	0.15	0.86
Tired	20.90 ± 20.32	35.79 ± 26.26	17.77 ± 19.68	20.89 ± 21.25	18.10 ± 21.32	29.66 ± 21.21	1.43	0.25
Tense	16.72 ± 15.55	6.61 ± 6.93	15.80 ± 16.54	7.41 ± 15.10	14.45 ± 17.03	5.21 ± 9.24	0.11	0.91
Energetic	59.07 ± 25.52	42.71 ± 28.06	56.03 ± 29.53	47.48 ± 29.58	59.86 ± 29.17	40.69 ± 29.64	1.35	0.26
Angry	0.82 ± 1.47	2.12 ± 3.71	1.82 ± 5.15	2.33 ± 6.30	1.04 ± 4.09	1.07 ± 3.09	0.61	0.55
SAS (1–5)								
Concentration	3.38 ± 0.62	3.10 ± 0.67	3.47 ± 0.63	3.37 ± 0.56	3.45 ± 0.78	3.31 ± 0.66	0.71	0.50
Calmness	4.00 ± 0.80	3.86 ± 0.92	3.93 ± 0.79	3.73 ± 0.91	3.79 ± 0.82	3.93 ± 0.75	3.40	0.04^*^
Fatigue	2.38 ± 0.94	2.62 ± 0.82	2.37 ± 0.89	2.43 ± 1.01	2.76 ± 0.83	2.66 ± 0.81	1.02	0.37
Visual perception	3.59 ± 0.87	3.41 ± 0.87	3.67 ± 0.92	3.43 ± 0.90	3.55 ± 0.74	3.35 ± 0.67	0.08	0.91

MoCA, the Montreal Cognitive Assessment; NSE, serum neuron-specific enolase; PPT, the Purdue Pegboard Test; VAMS-R, a revised version of the Visual Analog Mood Scale; SAS, self-assessment scale. *Indicates *p* < 0.05.

For the resting state EEG data, there was no detection of any epileptic activities in all three groups by the automated software or the clinical doctor. The skin-electrode interface temperature was in the safe range (range: 23.5–29.2°C; mean: 26.01°C). For the AEs results, all the subitems showed no significant differences among the three groups, except for the subitem of tingling. More details are given in Table 4.

**Table 4:** The statistical results of adverse effects (AEs).

Items	20 Hz group	70 Hz group	Sham group	*χ* ^2^	*p*
Itching	Mild: 7; moderate: 2; considerable: 1	Mild: 7	Mild: 9	6.78	0.34
Headache	Mild: 3; moderate: 3	Mild: 6; moderate: 2	Mild: 5; moderate: 1	2.02	0.73.
Burning	Mild: 3	Mild: 2; moderate: 1	Moderate: 2	2.27	0.69
Warmth	Mild: 6; moderate: 1	Mild: 7	Mild: 3 moderate: 1	1.88	0.76
Tingling	Mild: 4; moderate: 2; considerable: 1	Mild: 13; moderate: 2; considerable: 1	Mild: 2; moderate: 1; considerable: 1	14.49	0.03^*^
Metallic taste	Mild: 2	Mild: 2	Mild: 1	0.40	0.82
Fatigue	Mild: 7; moderate: 5; considerable: 2	Mild: 5; moderate: 2; considerable: 1	Mild: 8; moderate: 1; considerable: 1	6.24	0.40
Vertigo	Mild: 5; moderate: 2	Mild: 3; moderate: 1	Mild: 4	2.85	0.58
Nausea	Mild: 2	Mild: 2	Mild: 2	0.00	1.00
Phosphene	Mild: 1	None	None	2.06	0.36

^*^Indicates *p* < 0.05.

### 3.2 Efficacy evaluation

For the simple reaction time task, the ANOVA of the reaction time showed no significant main effect of the group [*F*(2, 82) = 0.459, *p* = 0.633, η^2^ = 0.011] but a significant main effect of the time [*F*(1, 82) = 10.349, *p* = 0.002, η^2^ = 0.112], indicating that a learning effect may exist after the stimulation for all three groups. The interaction effect of the group and the time was not significant [*F*(2, 82) = 1.373, *p* = 0.259, η^2^ = 0.032]. However, when including only the 70 Hz and sham groups, the ANOVA showed a marginal significance for the interaction effect of the group and the time [*F*(1, 55) = 3.729, *p* = 0.059, η^2^ = 0.063]. The t-tests showed a significant increase in the sham group [*t*(27) = −3.346, *p* = 0.002, *d* = 0.632] rather than the 70 Hz group [*t*(28) = −0.716, *p* = 0.480, *d* = 0.133; see Figure 1D].

In the one-increment task, the ANOVA of the reaction time showed no significant main effect of the group [*F*(2, 83) = 0.107, *p* = 0.899, η^2^ = 0.003] but a significant main effect of the time [*F*(1, 83) = 12.995, *p* = 0.001, η^2^ = 0.135]. Most importantly, the interaction effect of the group and the time was significant [*F*(2, 83) = 3.207, *p* = 0.046, η^2^ = 0.072], indicating differential effects among the three stimulation groups in the one-increment task. The t-tests showed a significant decrease in the 70 Hz and sham groups [70 Hz: *t*(28) = 2.091, *p* = 0.046, *d* = 0.338; sham: *t*(27) = 4.857, *p* = 0.000, *d* = 0.896], but not in the 20 Hz group [20 Hz: *t*(28) = 0.111, *p* = 0.913, *d* = 0.021; see Figure 1D]. We also assessed the baseline difference among the three groups of participants and repeated the analysis by assessing the planned contrasts between sham-20Hz and sham-70Hz for each outcome measure, focusing on pre-to-post differences. The results were shown in Supplementary Tables 4, 5.

## 4 Discussion

The current study assessed the safety and efficacy of a novel high-current TI electrical stimulation targeting M1 in healthy individuals. For the safety evaluation, there were no significant differences in the biochemical and neuropsychological measurements before and after the high-current TI electrical stimulation among the two active stimulation groups and the sham group. Meanwhile, the 20 and 70 Hz high-current TI electrical stimulation showed significant distinct effects in M1, especially in the one-increment task, where the 20 Hz stimulation significantly slowed down the RT performance compared with the 70 Hz and sham groups.

Theoretically, the parameter used in the current study is adhere to safety guidelines of transcranial electrical stimulation. Several factors influence the safety of transcranial electrical stimulation, including the electrode configuration, stimulation intensity, polarity, duration, electrode size, and charge density beneath the stimulation electrodes (Nitsche et al., 2003; Bikson et al., 2016; Antal et al., 2017). In the current study, the results of the computational modeling showed that the estimated brain current density was significantly lower than the threshold associated with potential brain injuries.

The results in the current study demonstrated that the application of the high-current TI electrical stimulation (intensity: 15 mA (zero-to-peak) in a single channel, carrier frequency: 20 kHz, envelope: 20/70 Hz, 30 min) to human was safe. The stimulation did not cause any neuronal apoptosis, motor impairment of hands, cognitive impairment, significant changes in emotional or psychological states, or epileptic discharges. The temperatures at skin-electrode interfaces remained within the safe range, indicating that there was no heating or skin burns. For the adverse reactions, only a minority of participants reported more than moderate discomfort. Although the general safety guidelines restrict tES intensity to 4 mA, this limitation is not due to the potential harm caused by higher currents to brain tissue (Bikson et al., 2016; Antal et al., 2017). In fact, the current density produced by tES ranging from 0–4 mA in the brain is significantly lower (one to two orders of magnitude) than the current density threshold of 6.3–13 A/m^2^ that is known to cause brain damage. The actual limiting factor for increasing current intensity is the sensation experienced by the skin. Our findings demonstrate that even a high current TI electrical stimulation of 15 mA does not result in brain tissue damage or physiological and neuropsychological harm to the subjects. Our results were consistent with previous findings with high current. By using high frequency tACS (5 kHz) theta burst protocol with intensities of up to 10 mA on motor cortex, six out of 17 healthy participants reported mild side effects (Kunz et al., 2017). By using 40-min tACS with a frequency of 77.5 Hz and a current of 15 mA on the forehead for 20 sessions, more than 95% of the participants completed the trial without unwanted stimulation-associated adverse sensations (Wang et al., 2022; Zhu et al., 2024).

The findings in the current study also demonstrated the distinct effects of 20 and 70 Hz high-current TI electrical stimulation in M1. In the one-increment task, the 20 Hz group exhibited a significant increase in reaction time (RT) post-stimulation compared to the 70 Hz and sham groups. The results align with previous research emphasizing the role of beta oscillations (20 Hz) in motor learning (Espenhahn et al., 2019, 2020; Ma et al., 2022). Our previous work indicated that 4 mA TI electrical stimulation at 20 Hz targeting the human M1 enhanced implicit motor learning (Ma et al., 2022), consistent with findings from tACS at 20 Hz (Krause et al., 2016). Conversely, the current study revealed an inhibitory effect. The contrasting effects may be attributed to the different current intensity (Moliadze et al., 2012; Batsikadze et al., 2013). On the other hand, previous research has indicated that TI electrical stimulation at 70 Hz can enhance simple movements and motor cortex excitability (Ma et al., 2022) and tACS at 70 Hz have shown improvements in motor speed and acceleration (Joundi et al., 2012; Moisa et al., 2016). Consistent with these findings, the current study observed enhanced RT in the simple reaction time (SRT) task following 70 Hz stimulation compared to the sham group, highlighting the functional segregation of beta and gamma frequency oscillations within M1.

Increasing current intensity in tES has been shown to be crucial for enhancing its effectiveness. Research in tACS has demonstrated that higher current intensities can lead to increased local field potentials in brain regions such as the hippocampus and amygdala (Shan et al., 2023), suggesting a potential for greater impact on brain activity. Consistent findings across various tACS and tDCS studies have further supported the positive effects of higher current intensities on evoked potentials in the human brain (Moliadze et al., 2012; Antonenko et al., 2019; Kasten et al., 2019; Steinmann et al., 2022). In contrast, research has shown that lower current intensities of TI may not produce significant effects on brain oscillations, as demonstrated in a study where 1 mA current intensity did not result in a notable regulatory effect on α brain oscillation (von Conta et al., 2022). This highlights the importance of considering and implementing higher current intensities in tES research and clinical applications to maximize the therapeutic benefits and efficacy of stimulation protocols.

Typically, elevating the overall intensity of tES increases the likelihood of co-stimulation of neighboring brain regions. However, TI has the advantage of higher focality compared to tDCS and tACS (Grossman et al., 2017; Huang and Parra, 2019; Rampersad et al., 2019), as neurons are unable to respond effectively to high frequencies (Palmer et al., 1999; Hutcheon and Yarom, 2000). Consequently, despite the higher overall intensity, the risk of co-stimulating neighboring brain regions is diminished with TI, which is required to be further evidenced in future research.

As a 15 mA (zero-to-peak) current intensity was used in the current study, a stimulation mode with a 50% duty cycle was implemented for safety considerations with a potential to achieve stronger regulatory effects. The 50% duty cycle reduced the duration of stimulation, leading to a decreased accumulation of electrical charge, thus lowering the risk of brain damage. Besides, research on tDCS and TI electrical stimulation with theta burst mode has indicated that discontinuous stimulation might have superior regulatory effects over conventional continuous stimulation (Tambini et al., 2017; Hermiller et al., 2019, 2020; Wessel et al., 2023). Given that the current study is a preliminary TI study with such a high current intensity, safety remains our primary concern. Future studies will explore the effect of the high-current TI electrical stimulation with the theta burst mode.

Unlike the 2,000 Hz carrier frequency used in previous TI electrical stimulation studies, a 20 kHz carrier frequency was used in the current study, mainly to alleviate the skin sensation of the participants. Based on our findings in the safety and efficacy, a new TI electrical stimulation protocol was proposed for its application in the intervention for diseases with movements. Safety and efficacy are required to be tested when the stimulation parameters are changed in the future.

The current study still has some limitations. The motivation of developing the TI electrical stimulation is to develop a non-invasive form of deep brain stimulation (DBS). One of the limitations is that M1 is a shallow brain region. The safety and efficacy of the high-current TI electrical stimulation on deeper brain regions need to be examined in future studies. DBS is markedly suprathreshold. Though the current study using a current of 15 mA (zero-to-peak) in a single electrode, the TI paradigm aligns more closely with subthreshold tACS studies utilizing kHz and amplitude-modulated techniques (Kunz et al., 2017). It is also valuable to include a direct low-frequency tACS control to provide a clearer assessment of whether the effects observed with TI are unique to its mechanism. Besides, NSE could measure the extent of brain injury, but could not accurately reflect the damage to M1. In future studies, structural magnetic resonance imaging (MRI) could be underwent to measure potential brain damage as a supplement. Immediate EEG recording was used to monitor seizures after high-current TI electrical stimulation, which was primarily intended to quickly identify acute adverse effects and ensure participant safety rather than detect its potential longer-term effects or online effects. Incorporating long-term and online EEG monitoring in future studies could provide a more comprehensive understanding of the safety and efficacy of high-current TI electrical stimulation. Unfortunately, we did not specifically evaluate the blinding process. Moving forward, we will make sure to include a formal assessment of blinding effectiveness in our future studies. Finally, the actual electric field distribution in the human brain of the high-current TI electrical stimulation remains unknown. Stereoelectroencephalogram may help uncover the strength of the current in the brain, especially in the deep regions.

## 5 Conclusion

The current study reveals that the high-current TI electrical stimulation is safe and effective on the primary motor cortex in humans. TI electrical stimulation beating with different oscillations exerted differential effects on the motor functions. By increasing the current intensity, it makes the novel form of electrical stimulation potentially more effective in stimulating the deeper brain regions, which requires to be further explored in the future.

## Data Availability

The original contributions presented in the study are included in the article/Supplementary material. Further inquiries can be directed to the corresponding authors.
