# Supplementary material for: The safety and efficacy of applying a high-current temporal interference electrical stimulation in humans
Source: Front Hum Neurosci. 2024 Nov 29;18:1484593. doi: 10.3389/fnhum.2024.1484593 (PMC11638170; doi:10.3389/fnhum.2024.1484593)
Supplement: Supplementary file 1 [file Data_Sheet_1.pdf]

Supplementary Material

The safety and efficacy of applying a high-current temporal interference stimulation in humans

Yan Wang[1,2,#], Ginger Qinghong Zeng[4,#], Mengmeng Wang[1,2], Mingsong Zhang[2,4], Chuangchuang Chang[2,4], Qiongwei Liu[2], Keqing Wang[2], Ru Ma[2], Ying Wang[2,6,*], and Xiaochu Zhang[1,2,3,4,5,*]

[1] School of Mental Health, Bengbu Medical University, Bengbu, Anhui 233000, China

[2] Division of Life Science and Medicine, University of Science & Technology of China, Hefei 230027, China

[3] Department of Psychology, School of Humanities & Social Science, University of Science and Technology of China, Hefei 230027, China

[4] Application Technology Center of Physical Therapy to Brain Disorders, Institute of Advanced Technology, University of Science & Technology of China, Hefei 230031, China

[5] Biomedical Sciences and Health Laboratory of Anhui Province, University of Science & Technology of China, Hefei 230027, China

[6] Anhui Provincial Stereotactic Neurosurgical Institute and Anhui Province Key Laboratory of Brain Function and Brain Disease Hefei 230027, China

[#] These authors contributed equally

1 Supplementary Methods

2.7 High-current temporal interference stimulation

The current density calculation formula for one pair of electrodes is as follows:

The current signal through each pair of electrodes is sinusoidal AC.

$$i = A \sin 2\pi f t;$$

$$i_1 = \frac{A}{7} \sin 2\pi f t;$$

$$i_2 = \frac{3A}{7} \sin 2\pi f t;$$

$$i_3 = \frac{5A}{7} \sin 2\pi f t;$$

i: current expression; i1, i2, i3: current expressions in the rising and falling stages; A: current intensity; f: AC frequency; t: single envelope time;

$$\mathbf{Q = R}\int_0^T dQ$$

$$= 2\mathrm{R}\int_0^t i_1^2 dt + 2\mathrm{R}\int_0^t i_2^2 dt + 2\mathrm{R}\int_0^t i_3^2 dt + 10\mathrm{R}\int_0^t i^2 dt;$$

$$\mathbf{I} = \sqrt{\frac{Q}{RT}};$$

$$\mathbf{J} = \frac{I}{S};$$

Q: energy generated in a stimulus cycle time; T: a stimulus cycle time; R: resistance value; I: effective current; J: current density; S: contact area between electrodes and scalp

Notably, The skin current density generated by high current TI is calculated here, according to previous studies, the ratio of skin current density to brain current density is 10:1 to 400:1 (Bikson et al., 2016), then the brain current density generated by high current TI can be calculated.

2.8 Validation of Safety and Efficacy Using Agar Tissue Phantom

The two-dimensional human brain Agar phantom in this experiment includes four concentric circles: scalp, skull, cerebrospinal fluid, and brain parenchyma (conductivity: scalp: 0.465 S/m, skull: 0.01S /m, cerebrospinal fluid: 1.65S /m, brain parenchyma: 0.2S /m) (Huang et al., 2017). The corresponding radii are 90 mm, 85 mm, 78 mm, and 71 mm (see Supplementary Figure 1A).

The NaCI/Agar concentration ratio is determined by calibration of the conductivity of the Agar ratio (see Supplementary Figure 1B), including the following steps:

1) Four different concentrations of NaCI/Agar solution were prepared (Agar: 30 g/L, NaCI: 1,2,4,6 g/L);
2) Cylindrical samples of different lengths (0.5, 1,2,4,6,8,10 cm) were prepared, and two metallic silver electrodes with the same area as the bottom of the sample were connected on both sides of the sample;
3) Use the function generator (JDS6600, JUNTEK, China) to apply 20000 Hz, 10 v AC voltage;
4) Calculate the conductivity of the Agar phantom. The calculation formula is as follows:

$$\boldsymbol{Z_{eff}} = \frac{\boldsymbol{V_2 R_S}}{\boldsymbol{V_1}};$$

$$\boldsymbol{Z_{eff}} = \frac{\boldsymbol{1}}{\boldsymbol{Y_{eff}}} = \boldsymbol{Z_{e1}} + \boldsymbol{Z_{sample}} + \boldsymbol{Z_{e2}};$$

Based on Ohm’s law, the Y_{eff} can be further expressed as:

$$\boldsymbol{Y_{eff}}(\boldsymbol{f}) = \frac{\boldsymbol{K_1}(\boldsymbol{f})}{\boldsymbol{L} + \boldsymbol{K_2}(\boldsymbol{f})};$$

$$\boldsymbol{K_1}(\boldsymbol{f}) = \boldsymbol{S} * (\boldsymbol{\sigma} + \boldsymbol{j2\pi f \varepsilon});$$

$$\boldsymbol{K_2}(\boldsymbol{f}) = \boldsymbol{K_1}(\boldsymbol{f}) * (\boldsymbol{Z_{e1}} + \boldsymbol{Z_{e2}});$$

The curve coefficients K_1 and K_2 can be obtained by nonlinear least squares fitting. The conductivity of the Agar phantom can be expressed as:

$$\boldsymbol{\sigma} = \boldsymbol{realpart}(\frac{\boldsymbol{K_1}}{\boldsymbol{S}});$$

V_1, V_2： the voltage at both ends of the sampling resistor and the Agar phantom sample; R_s: the resistance value of the sampling resistor; Z_{e1}， Z_{e2}， Z_{eff}: the electrode contact impedance and model

impedance, and the total impedance; Y_{eff}: the admittance value; f : the stimulation frequency; L: the sample length; ε : the dielectric constant of the sample.

5) The conductivity of four different concentrations of NaCI/Agar solution (Agar: 30g/L, NaCI: 1,2,4,6 g/L) was measured. Then, the conductivity distribution of Agar phantoms with different concentrations of NaCl was further determined by fitting curves (see Supplementary Figure 1C).

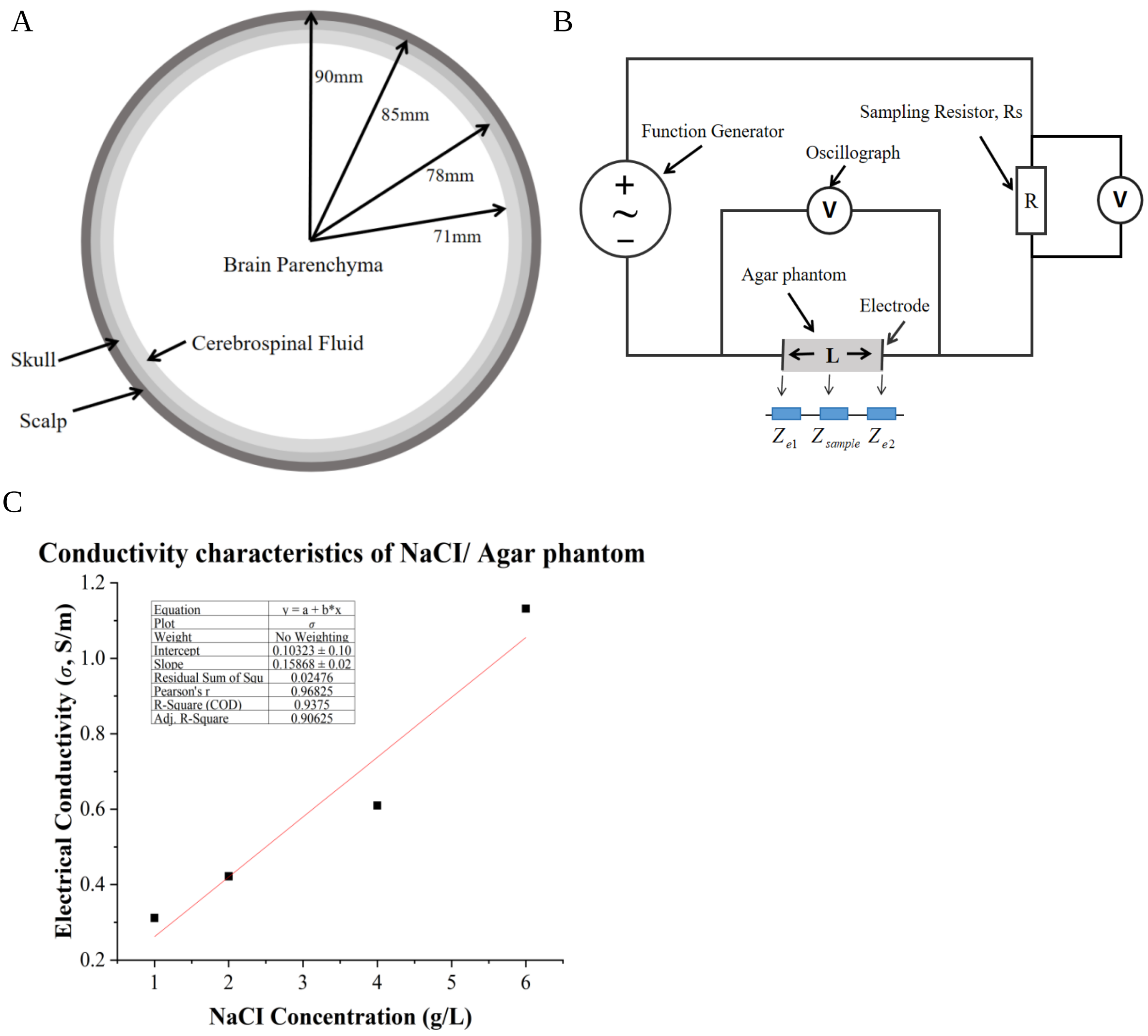

Supplementary Figure 1. (A) Geometric dimensions of the two-dimensional human brain Agar phantom. (B) Electrical conductivity measurement method of Agar phantom. (C) Electrical conductivity characteristics of Agar phantom.

High-current TI stimulation is applied to the Agar phantom. The electric field intensities under the four stimulation electrodes and the electric field intensities in different subcranial depths (1.5, 2, 2.5, 3, 4, 5, 6, 8cm) in the midline of brain parenchyma were measured by an oscilloscope (RIGOL, DHO4404, China) (see Supplementary Figure 2A). The electric field intensity at each position was measured three times to ensure the stability of the measurements. The current density is equal to the electric field strength multiplied by the conductivity. The waveform generated by the high-current stimulation was measured by the oscilloscope (see Figure 2B). The current density measured under the four electrodes are shown in Supplementary Table 1.

A

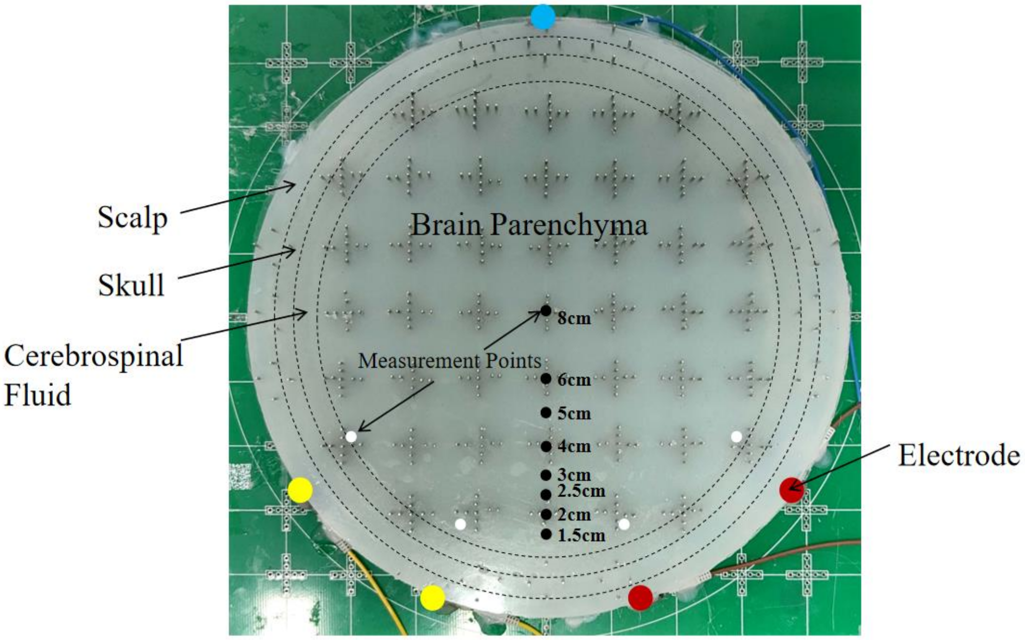

B

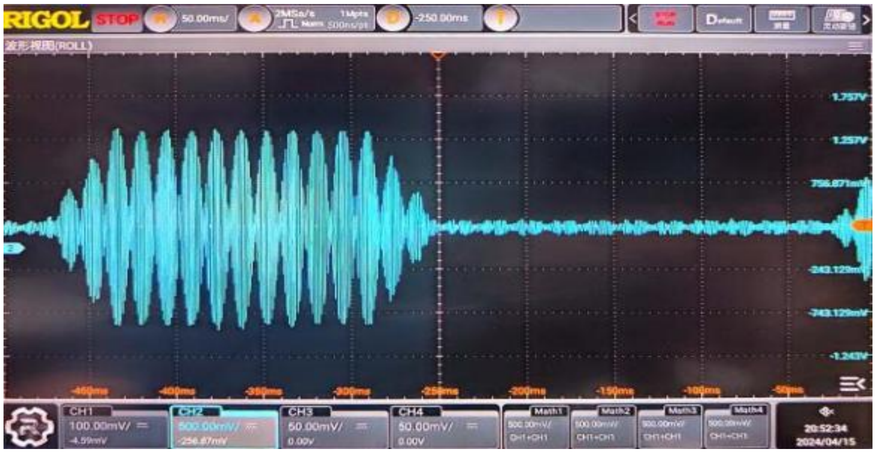

Supplementary Figure 2. (A) The electrode placement and measurement points in Agar phantom. Two pairs of electrodes are placed around the Agar phantom (one pair in red and the other pair in yellow, The electrodes are numbered 1,2,3,4 from left to right), with the blue ground electrode. The electric field intensities under the four

electrodes were measured at white points, meanwhile the electric field intensities at the subcranial part with different depths (1.5, 2, 2.5, 3, 4, 5, 6, 8cm) were measured at black points. (B) The waveform of the70 Hz high-current TI stimulation in the Agar phantom, stimulation and non-stimulation times were 229 ms, one stimulus consisted of 16 envelopes, and one envelope time was 14 ms.

Supplementary Table 1. The current density measured under the four electrodes

**Electrodes**	**Current density（A/m^2）**
electrode 1	1.85
electrode 2	1.63
electrode 3	1.75
electrode 4	1.75

2 Supplementary Results

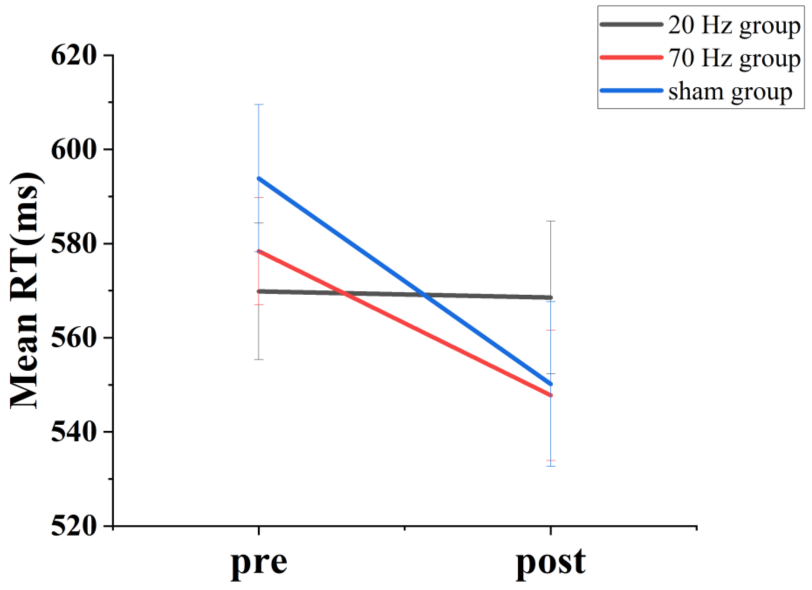

Supplementary Figure 3. Results of the mean reaction time in the one-increment task. The interaction effect of the group and the time was significant. RT is reaction time

Supplementary Table 2. The applications of temporal interference (TI) electrical stimulation in humans.

References	Subjects	Electrode type	Intensity (zero-peak)	F (kHz)	Δf (Hz)	Target	Outcomes
(von Conta et al., 2022)	Healthy subjects	Transcutaneous electrodes (5 cm × 5 cm)	0.5 mA	1	Individual alpha frequency	Occipital and parietal lobes	TI don't have an effect on human brain oscillations in the a-band
(Ma et al., 2022)	Healthy subjects	Circular transcutaneous electrodes (radius 10 mm)	1 mA	2	20/70	Primary motor cortex (M1)	TI has a significant promoting effect on human motor function;<br>The efficacy of TI has been validated in human brains for the first time;<br>Side effects occurring during TI stimulation were minor and tolerable.
(Piao et al., 2022)	Healthy subjects	Circular transcutaneous electrodes (radius 10 mm)	1 mA	2	20/70	Primary motor cortex (M1)	TI is safe and tolerable for humans in conditions similar to typical tES conditions.
(Wessel et al., 2023)	Healthy younger and older participants	Circular transcutaneous electrodes (radius 7 mm)	1 mA	2	100	Striatum (putamen, caudate and nucleus accumbens)	TI can noninvasively modulate striatal activity and improve motor learning in humans.
(Violante et al., 2023)	Human cadaver and healthy subjects	Transcutaneous electrodes with rounded corners (1.5 cm × 1.5 cm)	1-3 mA	2	5	The anterior /mid left hippocampus	TI can be steerably localized to the human hippocampus with minimal exposure of the overlying cortex;<br>TI can modulate hippocampal activity and enhance the accuracy of episodic memories in healthy humans;<br>Participants tolerated well the TI stimulation, there were no adverse

							effects and only a few incidences of mild common side effects.

F, carrier frequency of TI; Δf, envelop frequency.

Supplementary Table 3. The statistical results of main effects of the biochemical and neuropsychological measurements

**Measurements (Range or Unit)**	**Main effect of the time**			**Main effect of the group**		
	**F**	**p**	**η^2**	**F**	**p**	**η^2**
MoCA (0-30)	1.03	0.31	0.01	4.16	0.02*	0.09
NSE (ug/L)	13.40	0.00***	0.17	6.30	0.00**	0.16
PPT (times)						
Right hand	22.89	0.00***	0.21	0.29	0.75	0.01
Left hand	35.87	0.00***	0.30	0.30	0.75	0.01
Both hands	26.86	0.00***	0.24	0.01	0.99	0.00
Assembly	87.39	0.00***	0.51	1.64	0.20	0.04
VAMS-R (0-100)						
Sad	3.38	0.07	0.04	0.03	0.97	0.00
Confused	15.28	0.00***	0.16	0.47	0.63	0.01
Afraid	26.98	0.00***	0.25	0.05	0.95	0.00
Happy	0.31	0.58	0.00	0.25	0.78	0.01
Tired	12.91	0.00**	0.14	1.87	0.16	0.04
Tense	34.77	0.00***	0.30	0.20	0.82	0.01
Energetic	37.43	0.00***	0.32	0.04	0.96	0.00
Angry	1.10	0.30	0.02	0.49	0.62	0.02

SAS (1-5)						
Concentration	7.33	0.01**	0.08	0.73	0.49	0.02
Calmness	1.40	0.24	0.02	0.12	0.89	0.00
Fatigue	0.48	0.49	0.01	1.25	0.29	0.03
Visual perception	11.03	0.01**	0.12	0.13	0.88	0.00

Abbreviations: MoCA: the Montreal Cognitive Assessment; NSE: serum neuron-specific enolase; PPT: the Purdue Pegboard Test; VAMS-R: a revised version of the Visual Analog Mood Scale; SAS: self-assessment scale. * indicates p < 0.05, ** indicates p < 0.01, *** indicates p < 0.001.

Supplementary Table 4. The One-way ANOVA results of the difference of the demographic and pre-test results

**Measurements (Range or Unit)**	**F**	**p**
Year	0.63	0.54
Education level	1.53	0.22
Gender	0.08	0.92
MoCA (0-30)	2.66	0.08
NSE (ug/L)	4.39	0.02*
PPT (times)		
Right hand	0.04	0.96
Left hand	0.43	0.65
Both hands	0.02	0.99
Assembly	1.41	0.25
VAMS-R (0-100)		
Sad	0.02	0.98

Confused	0.06	0.94
Afraid	0.24	0.79
Happy	0.32	0.73
Tired	0.21	0.81
Tense	0.14	0.87
Energetic	0.15	0.86
Angry	0.32	0.73
SAS (1-5)		
Concentration	0.13	0.88
Calmness	0.50	0.61
Fatigue	1.83	0.17
Visual perception	0.14	0.87
Motor tasks (RT, ms)		
SRT	0.51	0.60
One-increment time Task	1.01	0.37

Abbreviations: MoCA: the Montreal Cognitive Assessment; NSE: serum neuron-specific enolase; PPT: the Purdue Pegboard Test; VAMS-R: a revised version of the Visual Analog Mood Scale; SAS: self-assessment scale. SRT: the simple reaction time task. * indicates $p < 0.05$, ** indicates $p < 0.01$, *** indicates $p < 0.001$.

Supplementary Table 5. The statistical results of pre-to-post differences between 20 Hz-sham and 70 Hz-sham

**Measurements (Range or Unit)**	**20 Hz-sham**		**70 Hz-sham**	
	**t**	**p**	**t**	**p**
MoCA (0-30)	0.05	0.96	0.33	0.75
NSE (ug/L)	-1.5	0.13	-0.86	0.40

PPT (times)				
Right hand	0.31	0.76	-0.82	0.42
Left hand	-1.91	0.07	-0.89	0.38
Both hands	-0.86	0.40	-0.53	0.60
Assembly	-0.43	0.67	-1.74	0.09
VAMS-R (0-100)				
Sad	1.10	0.28	1.00	0.33
Confused	-0.05	0.96	1.21	0.24
Afraid	1.34	0.19	1.74	0.09
Happy	0.31	0.76	0.69	0.49
Tired	-0.30	0.77	1.60	0.12
Tense	0.35	0.73	0.06	0.95
Energetic	-0.24	0.82	-0.76	0.45
Angry	-0.60	0.56	0.50	0.63
SAS (1-5)				
Concentration	0.75	0.46	-0.21	0.84
Calmness	1.98	0.06	1.98	0.06
Fatigue	-1.54	0.13	-0.82	0.42
Visual perception	-0.20	0.85	0.30	0.77
Motor tasks (RT, ms)				
SRT	-0.35	0.73	1.29	0.21
One-increment time Task	-3.26	0.00**	-1.14	0.27

Abbreviations: MoCA: the Montreal Cognitive Assessment; NSE: serum neuron-specific enolase; PPT: the Purdue Pegboard Test; VAMS-R: a revised version of the Visual Analog Mood Scale; SAS: self-assessment scale. SRT: the simple reaction time task. * indicates $p < 0.05$, ** indicates $p < 0.01$, *** indicates $p < 0.001$.

3 References

Bikson, M., Grossman, P., Thomas, C., Zannou, A. L., Jiang, J., Adnan, T., et al. (2016). Safety of Transcranial Direct Current Stimulation: Evidence Based Update 2016. *Brain Stimul.* 9, 641–661. doi: 10.1016/j.brs.2016.06.004

Huang, Y., Liu, A., Lafon, B., Friedman, D., Dayan, M., Wang, X., et al. (2017). Measurements and models of electric fields in the in vivo human brain during transcranial electric stimulation. *Brain Stimul.* 10, e25–e26. doi: 10.1016/j.brs.2017.04.022

Ma, R., Xia, X., Zhang, W., Lu, Z., Wu, Q., Cui, J., et al. (2022). High Gamma and Beta Temporal Interference Stimulation in the Human Motor Cortex Improves Motor Functions. *Front. Neurosci.* 15. doi: 10.3389/fnins.2021.800436

Piao, Y., Ma, R., Weng, Y., Fan, C., Xia, X., Zhang, W., et al. (2022). Safety Evaluation of Employing Temporal Interference Transcranial Alternating Current Stimulation in Human Studies. *Brain Sci.* 12, 1194. doi: 10.3390/brainsci12091194

Violante, I. R., Alania, K., Cassarà, A. M., Neufeld, E., Acerbo, E., Carron, R., et al. (2023). Non-invasive temporal interference electrical stimulation of the human hippocampus. *Nat. Neurosci.* 26, 1994–2004. doi: 10.1038/s41593-023-01456-8

von Conta, J., Kasten, F. H., Schellhorn, K., Ćurčić-Blake, B., Aleman, A., and Herrmann, C. S. (2022). Benchmarking the effects of transcranial temporal interference stimulation (tTIS) in humans. *Cortex* 154, 299–310. doi: 10.1016/j.cortex.2022.05.017

Wessel, M. J., Beanato, E., Popa, T., Windel, F., Vassiliadis, P., Menoud, P., et al. (2023). Noninvasive theta-burst stimulation of the human striatum enhances striatal activity and motor skill learning. *Nat. Neurosci.* 26, 2005–2016. doi: 10.1038/s41593-023-01457-7
